# Pazolimus: pazopanib plus sirolimus following progression on pazopanib, a retrospective case series analysis

**DOI:** 10.1186/s12885-016-2618-1

**Published:** 2016-08-08

**Authors:** D. Katz, Y. Azraq, F. Eleyan, S. Gill, T. Peretz, O. Merimsky

**Affiliations:** 1 Sharett Institute of Oncology, Hadassah-Hebrew University Medical Center, Jerusalem, Israel; 2 Department of Radiology, Hadassah-Hebrew University Medical Center, Jerusalem, Israel; 3 Boston University School of Medicine, Boston, USA; 4 The Unit of Bone and Soft Tissue Oncology, Division of Oncology, Tel-Aviv Sourasky Medical Center, Tel-Aviv, Israel; 5 Affiliated with Sackler School of Medicine, Tel-Aviv University, Tel-Aviv, Israel

**Keywords:** Sarcoma, Solitary fibrous tumor, Chondrosarcoma, Pazopanib, Sirolimus, VEGF, mTOR, Tyrosine kinase inhibitor, Resistance

## Abstract

**Background:**

To explore the activity of pazopanib (P) + sirolimus (S) in patients who progressed after previous clinical benefit on pazopanib.

**Methods:**

Eight patients with progressing metastatic high grade soft tissue sarcoma (STS) whose disease advanced on P following a response duration of at least 4 months were offered re-challenge of P supplemented by off-label S and a single patient with progressing metastatic chondrosarcoma was offered the combination as compassionate treatment. Patients were treated in two centers: Hadassah Medical Center and Tel Aviv Medical Center. Patients received oral P 200–600 mg once a day supplemented by S 3–4 mg taken separately, 12 h after the P dose.

**Results:**

Patients received treatment from December 2012 to February 2016. Four progressed on the combination and their treatment was terminated. Two patients were undergoing treatment when data was summarized. Best Response Evaluation Criteria in Solid Tumour (RECIST) responses were: one partial response (PR), four stable disease (SD), and four progressive disease (PD), corresponding to five PR and four PD on the Choi criteria. Median progression free survival was 5.5 months (range 4–17).

**Conclusions:**

Our series showed that the combination of P + S has activity in STS patients selected by previous response to P and in a patient with chondrosarcoma, suggesting this can serve as a mechanism to reverse resistance to P and extend the chemotherapy-free window.

## Background

Soft tissue sarcoma (STS) treatment arsenal included until 2012 only chemotherapies given as single agents or in combination [1]. Many of these chemotherapy protocols produce serious, intolerable toxicities such as pancytopenia, alopecia and nephrotoxicity. Pazopanib (P), a vascular endothelial growth factor (VEGF) receptor inhibitor was granted approval by the FDA and EMA for the treatment of STS patients in second line and beyond. According to the registration trial, the PALLETE trial, P increased mean progression free survival (PFS) by 3 months compared to placebo with a manageable toxicity profile comprised mainly of fatigue, diarrhea, hypertension and hair hypopigmentation that differs significantly from that of chemotherapy [2].

Other classes of targeted drugs were evaluated in STS but none possessed convincing clinical benefit. One of these classes consists of mammalian target of rapamycin (mTOR) inhibitors, a class of drugs with anti-proliferative effects supporting their role as anti-cancer agents [3]. Sirolimus (S) was the first drug in the class to be evaluated as an anti-cancer agent and remains the most convenient because of its low price and favorable toxicity profile [4]. S has been tried in a sample of sarcoma patients alone and in combination with chemotherapeutic agents such as cyclophosphamide and gemcitabine with intriguing results [5–9].

However, much of the recent research has been performed using newer patented agents within this family, such as ridaforolimus and everolimus. Ridaforolimus, a new mTOR inhibitor analogue, was the only compound to be evaluated as a maintenance agent in metastatic STS. The study demonstrated a PFS increase of 3.1 weeks [10]. Although this study exhibited tumor growth control, it lacked the clinical significance to allow approval for use by any drug legislation agency. Everolimus was studied in combination with sorafenib, a VEGF receptor inhibitor among others, in patients with unresectable osteosarcoma which showed a 45 % PFS but fell short of the target endpoint 50 % 6-month PFS and was therefore considered negative [11].

As responses to pazopanib are rarely durable and resistance develops in the absence of additional evidence based target therapies, chemotherapy is recommended. However, reversal of resistance may as well be sought, especially in those cases where pazopanib has been well tolerated offering advantageous quality of life over chemotherapy [12–14]. Emerging preclinical and clinical data for multikinase and mTOR inhibitors relies on the mechanistic hypothesis that the combination blocks angiogenesis at two different points in the signaling pathway and suggests that their concomitant administration after progression on pazopanib has the potential to offer further disease stabilization and prolong the chemotherapy-free window [15, 16].

Here we report on a retrospective series of eight unresectable metastatic advanced STS patients and one chondrosarcoma patient treated with P + S.

## Methods

Patients with progressing metastatic unresectable high grade STS, whose disease advanced on P following a response duration of at least 4 months were offered re-challenge of P supplemented by off-label S in two medical centers; Hadassah Medical Center and Tel Aviv Medical Center. A single patient with progressing unresectable metastatic chondrosarcoma resistant to chemotherapy was offered the combination as compassionate treatment. All patients were in good performance status with an ECOG 0–1. Patient data collection was initiated following local IRB approval.

### Treatment schedule and evaluation

Patients received oral P 200–600 mg once a day supplemented by S 3–4 mg taken separately, 12 h after the P dose. In those cases in which serum S levels were measured 7–14d after treatment was started, S dose was adjusted for a serum level of 15–20 ng/ml. Drug dosage was reduced according to toxicity. Prior to the initiation of the combination therapy, chest, abdomen and pelvis contrast CT or PET-CT were done and repeated at 6–8 w intervals and after 6 months at 12 w intervals. Patients with response or stable disease (SD) continued treatment until disease progression (PD). Blood tests were repeated bi-weekly in the first month and monthly thereafter. Toxicity was recorded at each clinic visit and summarized in Table 1.

**Table 1:** Patient characteristics and clinical and molecular findings

Patient #	Pathology	Primary site/Recurrence	DFI from surgery to relapse (mos)	Number of lines before P	Duration of P treatment (mos)	Duration of P + S treatment (mos)	Best RECIST response	Best Choi response	P dose (mg)	S dose (mg)	Procedures while on P + S	Toxicity	Status	Genomic profile by FoundationOne
1	SFT	Lung/Lung + heart	10	0	4	2	PD	PD	400	4			AWD	
2	SFT	Pleura/Duadenum + Spleen + liver	13	0	8	2	PD	PD	600	4		Lethargy	Dead	NAB2-STAT6 fusion
														TP53 P278S
														AXIN1 A740T
														BRD4 truncation exon 8
3	UPS	Limb/Lung	24	3	22	2	PD	PD	800	3			Dead	
4	UPS	Gluteus/Lung + pleura	41	3	6	5	PR	PR	200	4		Glucose intolerance-metformin	Dead	
5	ULMS	Uterus/Lung	13	2	8	16 on going	SD	PR	400	3	Metastasectomy and SBRT	Sub-febrile fever, glu intolerance-metformin	AWD	ALK IGFGP5-ALK fusion
														TSC2 splice site*
														CDKN2A loss
														TP53 L330P
6	LMS	Limb/Lung	14	1	28	5	SD	PR	400	3		Bowel perforation	AWD	
7	DSRCT	Retroperitoneum	0	3	12	11	SD	PR	200	3		Proteinuria	Dead	
8	UUS	Uterus/Lung + retroperitoneum	3	1	4	1	PD	PD	400	4			Dead	
9	Chondrosarcoma grade II	Limb/Lung	47	1	0	3 on going	SD	PR	400	4			AWD	CKS1B amplification
														MEF2C amplification

*see Fig. 3

### Outcome evaluation

Response was assessed according to the Response Evaluation Criteria in Solid Tumors (RECIST) (version 1.1) and Choi criteria. According to Choi criteria, response is based on both a minimum of a 10 % reduction in size and a 15 % reduction in density. Progression was defined as new lesions, an increase in ≥10 % in tumor size without meeting any criteria for a PR according to tumor density/contrast enhancement, or an increase ≥15 % in tumor density/contrast enhancement. PFS was calculated from the first date of treatment to the date of documented progression according to RECIST. Overall survival was computed from first date of treatment to the date of death or last date of follow up.

## Results

Between December 2012 to February 2016, nine patients received P + S in combination. Four patients progressed on the combination and their treatment was terminated. Two patients were undergoing treatment when we summarized the data.

Eight patients (five females); 2 solitary fibrous tumor (SFT), 2 undifferentiated pleomorphic sarcoma (UPS), 1 uterine leiomyosarcoma (ULMS), 1 leiomyosarcoma (LMS), 1 desmoplastic small round cell tumor (DSRCT), 1 undifferentiated uterine sarcoma (UUS) between the ages of 36–74 received P + S in combination, following escape from response to P of at least 4 months and one patient with grade II metastatic chondrosarcoma received the combination without initial P (Table 1). Patients ages ranged between 20 and 74 years old.

**Fig. 1:**
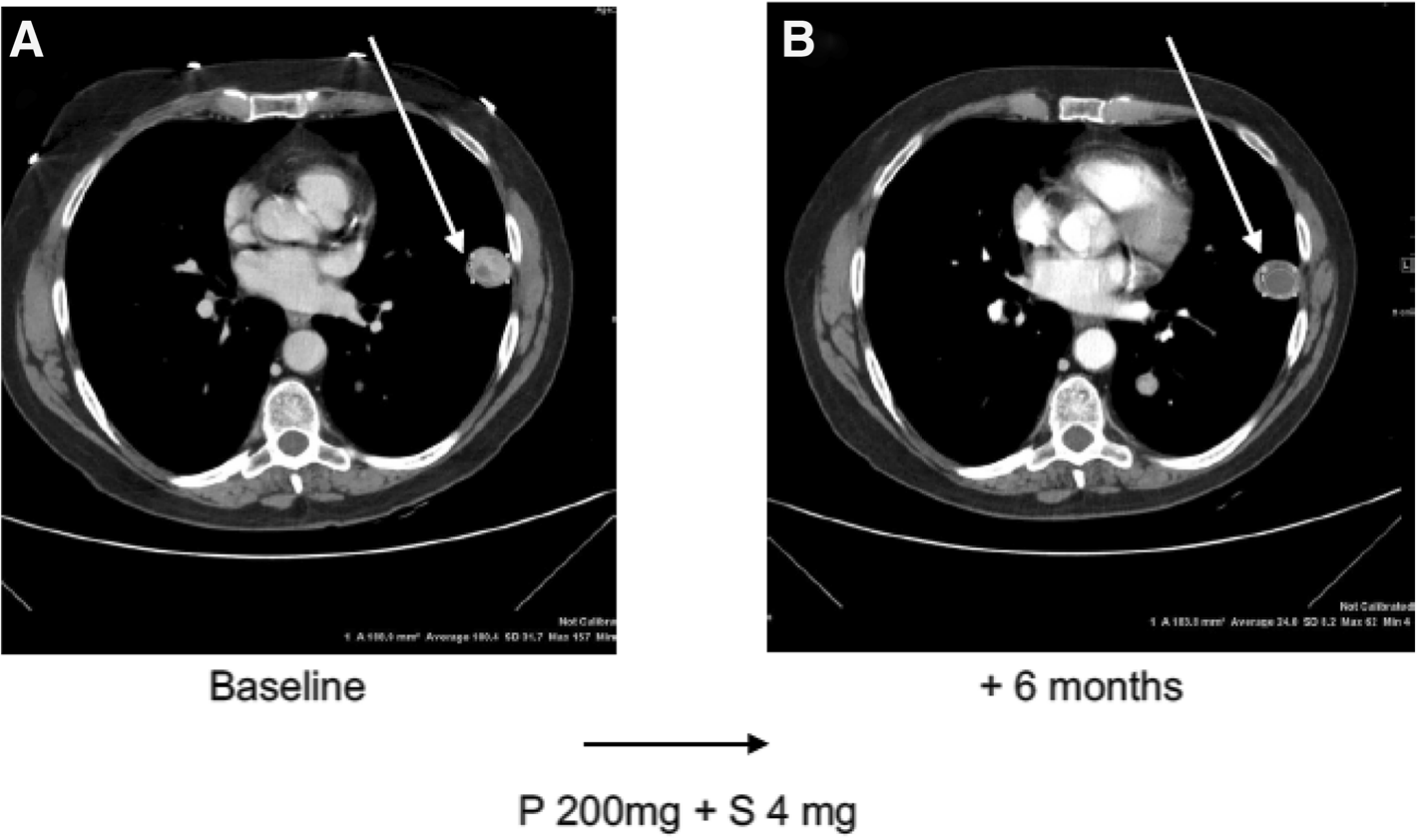
Response to P + S combination therapy. Computed tomography (CT) scan (arterial phase after contrast medium) of the chest. (**a**) Baseline (**b**) Six months after starting P + S combination therapy. Arrows indicate the response observed in the intrathoracic lesion, marked by decrease in tumor density

Five patients (5/8) benefited from the combination. Best RECIST responses were one PR (patient #4 with UPS), four SD and four PD. Median PFS was 5.5 m (range 4–17). Two patients are still on treatment (patients 5 and 9). Four patients are alive. Patient #5 underwent two consecutive resections of three lung metastases (right middle lobe, subpleural and left lower lobe) after completing 6 m of P + S (P 200 mg + S 4 mg) with SD as best response according to RECIST and PR according to Choi (see Fig. 1). Prior to treatment initiation, she was rapidly progressing on P single agent. Baseline CT demonstrated a new right lung lesion compared with CT 6 weeks prior. On Pathology the RML lesion was completely fibrotic, the left sub-pleural lesion consisted of extensive necrosis with scarce residual sarcoma cells and the left lower lobe lesion was composed mainly of viable sarcoma tissue. The patient’s treatment was discontinued following the oligo-metastasectomy. Three months later, chest CT demonstrated a new RML lesion and treatment with P + S was resumed (P 200 mg + S 4 mg). Following 7.5 months of stable disease, stereotactic body radiotherapy (SBRT) was administrated to the single right middle lobe lesion while the patient’s treatment continued.

There was one serious adverse event of small bowel perforation after 5 months on treatment, which was surgically treated with complete recovery. On pathology, a necrotic metastasis was present on the wall of the small bowel at the site of perforation. Additionally, two patients developed hyperglycemia compliant to metformin, one patient complained of lethargy. Overall, treatment was very well tolerated without added side effects.

## Discussion

We assessed the efficacy of the combination P + S in a series of eight patients with unresectable metastatic high grade STS and one patient with grade II chondrosarcoma. Currently, there are no evidence-based molecular predictive markers for response to tyrosine kinase inhibitors [17]. In order to optimize the chance of response, we enriched the treatment group with patients who had already shown benefit from P alone and escaped response, with the hope that this strategy would reverse resistance and delay the use of cytotoxic compounds. This study was not designed to delineate efficacy by STS subtype, but rather serve as a proof of concept that P + S is an effective way to combat resistance to P and prolong PFS. Out of the nine patients, four had S levels measured 7–14d after treatment was started and S dose was adjusted for a serum level of 15–20 ng/ml. However, since three patients responded despite not having their levels adjusted, we cannot determine that there is a correlation between blood level and response.

After the addition of S, we noticed additional limited toxicity. Only one patient developed a serious adverse event (SAE) (small bowel perforation) necessitating hospitalization and surgery with complete recovery. Of mention, the toxicity observed in this patient was related to the efficacy of the combination on the tumor, which was located on the wall of the small bowel. Less serious side effects developed in three patients; two patients developed hyperglycemia controlled by metformin and one patient developed a drug fever, which resolved over time. Overall, most patients tolerated the addition of S well. With the exception of the SAE above, there were no additional hospital admissions due to side effects from therapy. In terms of activity, 56 % (5/9) of the patients had a PFS of at least 5 months from the initiation of combination therapy. Following progression on the combination therapy, all STS patients received an additional line of chemotherapy.

**Fig. 2:**
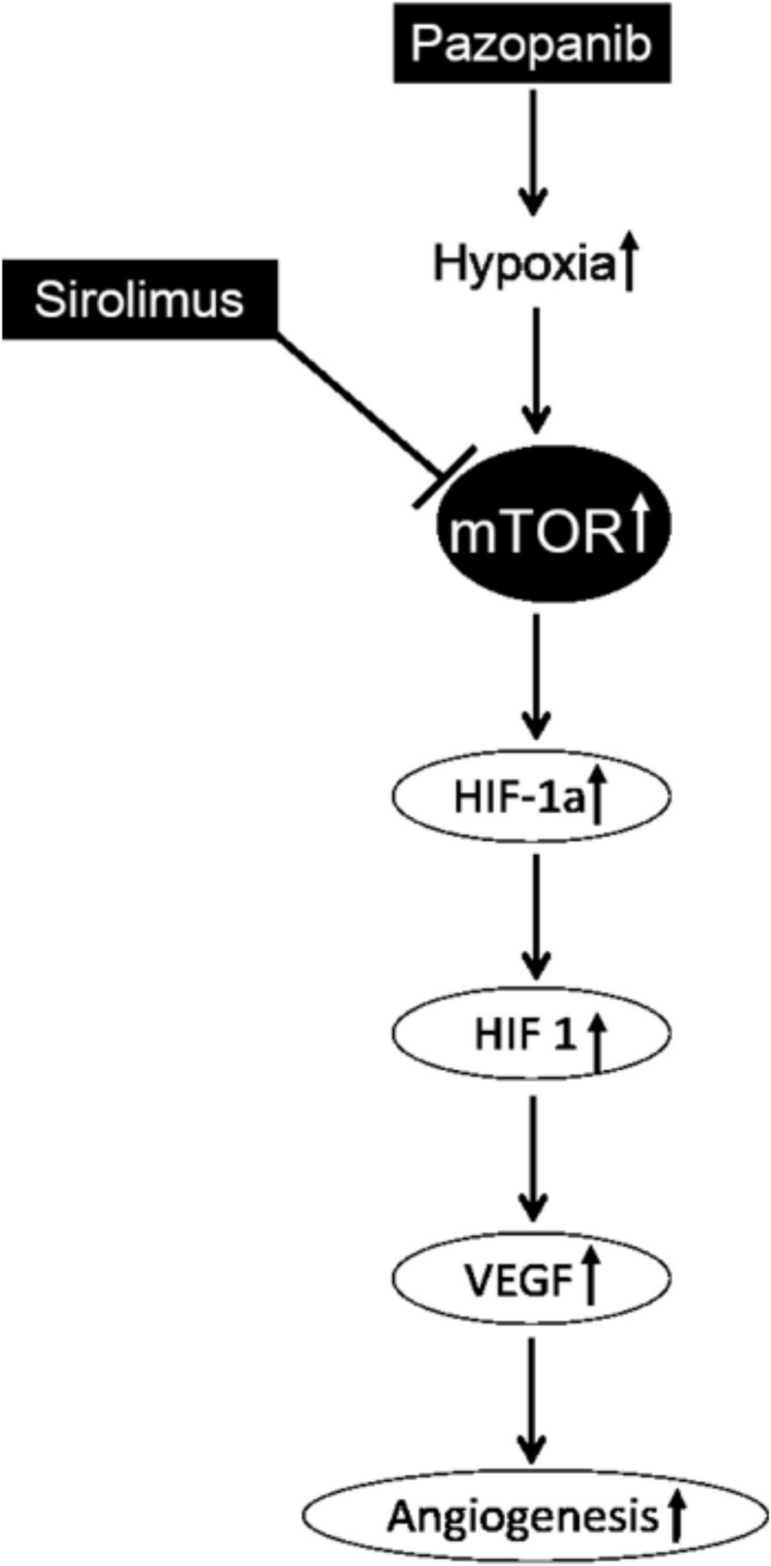
Compensatory VEGF overexpression. Initiation of pazopanib leads to a decrease in angiogenesis and development of hypoxia. Hypoxia causes an increase in HIF-1a levels, leading to increased production of target genes including VEGF. mTOR inhibition may stop this compensatory increase

We proposed supplementing P with S for various reasons. S has a favorable toxicity profile, its levels can be monitored, and preclinical and clinical data suggests that the combination with a VEGF receptor tyrosine kinase inhibitor (TKI) may potentially reverse resistance [8, 11, 15, 16, 18]. Pazopanib is a multikinase inhibitor that blocks the VEGF and PDGF receptors. However, acquired resistance to P eventually develops [12, 13, 19]. There are several potential mechanisms for the development of resistance; the most relevant being a compensatory increase in VEGF levels. As P blocks the VEGF receptor, hypoxia develops secondary to the negative effects of the receptor blockade on angiogenesis, leading to regression of blood vessels and an increase in HIF-1a levels via the mTOR pathway [18]. HIF-1a upregulates production of target genes including VEGF in the tumor microenvironment (Fig. 2). mTOR inhibition offsets the production of VEGF through complementary inhibition of the PTEN-AKT-mTOR pathway [20].

Another important adaptive mechanism of resistance to P is upregulation of fibroblast growth factor 2 (FGF2) [21]. FGF activates the FGF receptor inducing angiogenesis through the mTOR signaling pathway, along with several other pathways. Abrogating the mTOR pathway ameliorates angiogenesis induced by FGF2 [22]. On a different note, platelet derived growth factor receptor a (PDGFRa) was found to be activated by mTOR inhibition [22]. Therefore, combining S with an PDGFR inhibitor such as P may overcome potential resistance [23]. Furthermore, in the phase III SUCCEED trial designed to assess efficacy of single agent oral ridaforolimus on patients with STS, there was proof of tumor growth control, albeit clinically insignificant. The results of this trial support the use of combination therapy with other signaling inhibitors to overcome the activation of possible intracellular compensatory signaling pathways [10].

The rationale for adding mTOR inhibition to reverse resistance is not novel. The mTOR inhibitor everolimus was combined with an aromatase inhibitor exemestane in breast cancer patients who progressed while receiving an aromatase inhibitor. The results showed that adding everolimus increased PFS by 4.1 months [24]. In breast cancer, resistance to endocrine therapy is mediated through mTOR-induced phosphorylation of estrogen receptors and the addition of everolimus disrupts this phosphorylation and resulting resistance [25, 26]. An additional study evaluated the effect of the multikinase inhibitor, sorafenib, and the mTOR inhibitor, everolimus, in patients with metastatic osteosarcoma. The clinical benefit rate (CBR) at 6 months was 45 % with combination therapy, while another phase II study in a similar population showed a CBR of 29 % at 6 months for sorafenib alone [11, 15]. The study suggests that in osteosarcoma, resistance to sorafenib is mediated, at least partly, through the mTOR pathway. Sorafenib suppresses the mTORC1 pathway but simultaneously activates mTORC2 which promotes tumor progression. Adding the mTOR inhibitor everolimus overcomes this resistance. Regardless of the type of cancer, mTOR is a universal mediator of protein synthesis affecting angiogenesis and proliferation and mechanistically its inhibition may circumvent resistance [27].

**Fig. 3:**
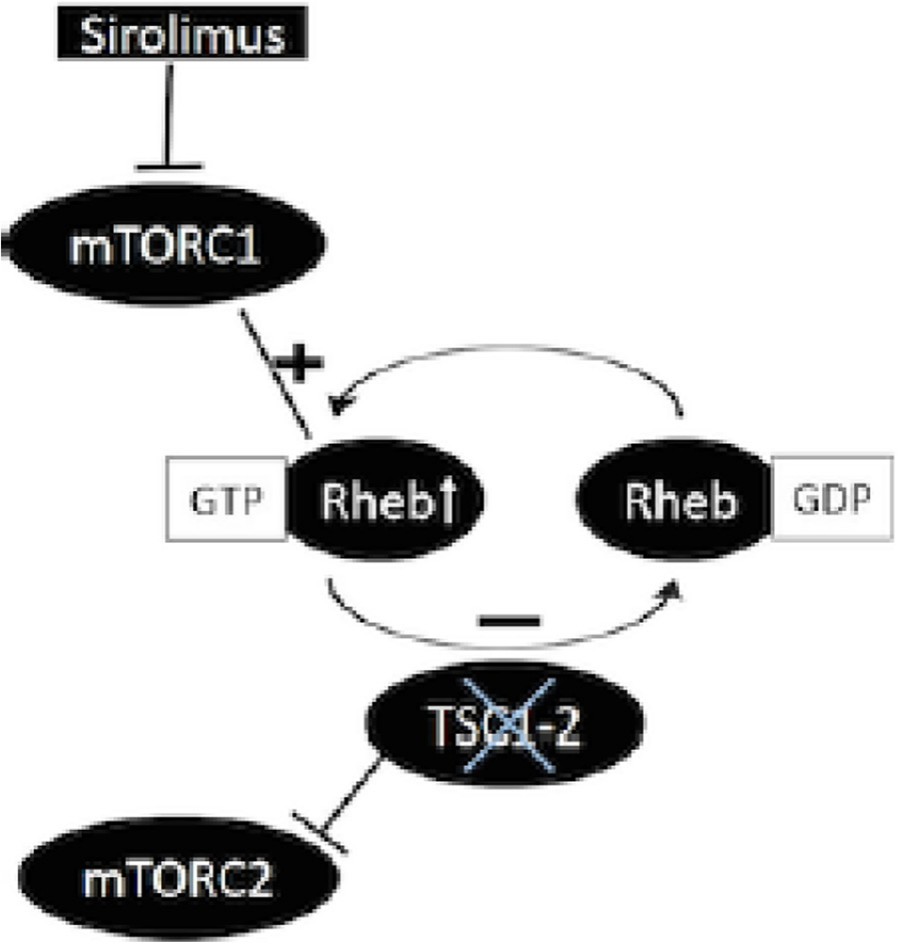
TSC1-2 complex activation and effect on mTOR pathway. TSC1-2 forms a complex with the GTPase domain of Rheb, converting it to its inactive, Rheb-GDP form. A loss of function mutation in TSC1-2 leads to increased levels and unopposed action of Rheb-GTP on mTORC1, leading to constant cell growth. Additionally, loss of function of TSC1-2 hinders mTORC2 activation

Patient #5 exhibited an “exceptional” response to P + S treatment. This patient underwent next generation sequencing (NGS) using the Foundation One test which showed a mutation in TSC2 splice site 2545. TSC2 is part of the TSC1-TSC2 complex, which inhibits mTORC1 through its Rheb-GAP activity. When the complex is active, the levels of Rheb-GTP decrease, inactivating mTORC1 and blocking its cell growth promotion (see Fig. 3). A mutation in TSC1 or TSC2 can lead to loss of function and constant activation of the mTORC complex [28]. This patient had a specific TSC2 mutation that had not been described in malignant tissue in COSMIC as of February 2016. However, it is known that TSC2 splice site alteration affects exon 22 and causes protein truncation [29]. This leads to inactivation of the GTPase domain of the TSC1-2 complex, leading to constant downstream activation of mTORC1 and cell growth.

**Fig. 4:**
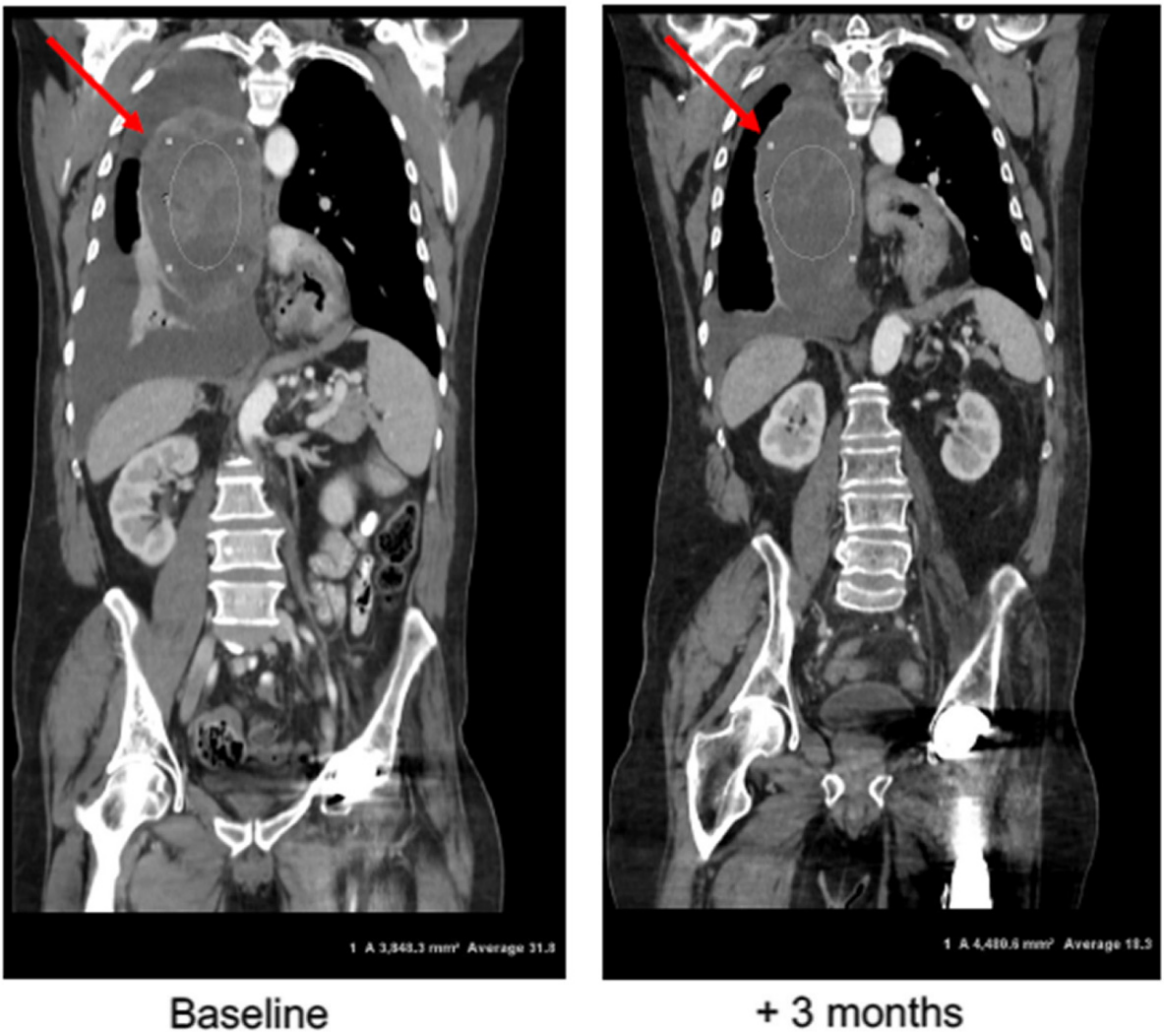
Response to P + S combination therapy in grade II paratracheal chondrosarcoma. Computed tomography (CT) scan. Three months after starting P + S combination therapy a response was observed in the form of tumor liquidification and stabilization of disease

We included one patient with a paratracheal unresectable grade II chondrosarcoma (#9) who had failed docetaxel in combination with gemcitabine. The P + S was offered as compassionate therapy given the lack of additional treatment options. The patient exhibited a rapid clinical response to the combination with resolution of chest pain, shortness of breath, and no re-accumulation of a pleural effusion that had previously been tapped. CT findings three months later confirmed the clinical improvement showing liquidification of the tumor with stabilization of disease (see Fig. 4). The patient’s P + S treatment is ongoing.

As previously mentioned, this study was not intended to evaluate efficacy by sarcoma subtype. However, two patients with metastatic SFT progressed on the combination therapy. In a study that evaluated P efficacy in SFT, a lower level activity was reported for P compared to sumitinib and bevacizumab plus temozolomide. This lower level of response was supported by preclinical data. Therefore, it is possible that progression on the combination seen in our study suggests real resistance within this subtype [19].

As seen with P alone, disease stabilization as defined by RECIST is the most frequent response in this series. However, Choi criteria assesses a change in density as well as size of the target lesions and therefore appears to be a better predictor of clinical response [30]. In our sample, all of the patients with SD by RECIST were confirmed to exhibit PR according to Choi.

Our clinical data, even if retrospective and on a small heterogenic group of patients, confirms that P + S was active in more than half of the patients. However, a potential limitation of this study is that response to single agent S following resistance to P was not tested. It is possible the patients in this study who benefited from P + S could have also benefited from S alone. However, combination P + S did not add significant additional toxicity, with its only downside being the additional cost. A future randomized control trial comparing P + S to S alone following resistance to P may further elucidate whether combination therapy is necessary. Because the arsenal of treatments in sarcoma is limited, any benefit observed by this combination therapy should be further investigated.

## Conclusion

Our series showed that the combination of P + S has activity in STS and chondrosarcoma patients selected by previous response to P. The goal of therapy for patients with metastatic sarcoma is to prolong life and palliate symptoms. Thus the favored approach remains to use less toxic drugs. P is currently the only approved targeted small molecule in second-line and beyond treatment in STS with a favorable toxicity profile which differs greatly from that of chemotherapy. Resistance to P eventually develops and the addition of S serves to prolong the chemotherapy-free window. This retrospective series proposes to enhance the therapeutic landscape of STS patients. We suggest that the current results serve as proof of concept for the use of combination P + S after escape from P and should be explored prospectively in a large randomized control trial to evaluate the efficacy of combination therapy in different sarcoma subtypes.

## Abbreviations

CBR, clinical benefit rate; DSRCT, desmoplastic small round cell tumor; FGF2, fibroblast growth factor 2; LMS, Leiomyosarcoma; mTOR, mammalian target of rapamycin; NGS, next generation sequencing; P, pazolimus; PD, progressive disease; PDGFRa, platelet derived growth factor receptor a; PFS, progression free survival; PR, partial response; RECIST, best response evaluation criteria in solid tumour; S, sirolimus; SAE, serious adverse event; SBRT, stereotactic body radiotherapy; SD, stable disease; SFT, solitary fibrous tumor; STS, soft tissue sarcoma; TKI, tyrosine kinase inhibitor; ULM, uterine leiomyosarcoma; UPS, undifferentiated pleomorphic sarcoma; UUS, undifferentiated uterine sarcoma; VEGF, vascular endothelial growth factor
